# Measuring mobility in older hospital patients with cognitive impairment using the de Morton Mobility Index

**DOI:** 10.1186/s12877-018-0780-9

**Published:** 2018-04-23

**Authors:** Tobias Braun, Christian Grüneberg, Christian Thiel, Ralf-Joachim Schulz

**Affiliations:** 1 Hochschule für Gesundheit (University of Applied Sciences), Department of Applied Health Sciences, Physiotherapy Program, Bochum, Germany; 2 University of Cologne, Medical Faculty, Cologne, Germany; 3 St. Marien-Hospital, Department of Geriatric Medicine, Cologne, Germany

**Keywords:** Mobility limitation, Outcome assessment, Geriatric assessment, Psychometrics, Rehabilitation, Physiotherapy, de Morton Mobility Index, Dementia

## Abstract

**Background:**

Mobility is a key outcome in older patients with cognitive impairment. The de Morton Mobility Index (DEMMI) is an established measure of older people’s mobility that is promising for use in older patients with cognitive impairment. The aim of this study was to examine the DEMMI’s psychometric properties in older patients with dementia, delirium or other cognitive impairment.

**Methods:**

This cross-sectional study was performed in a geriatric hospital and includes older acute medical patients with cognitive impairment indicated by a Mini Mental State Examination (MMSE) score ≤ 24 points. A Rasch analysis was performed to check the DEMMI’s unidimensionality. Construct validity was assessed by testing 13 hypotheses about expected correlations between the DEMMI and outcome measures of similar or related constructs, and about expected differences of DEMMI scores between groups differing in mobility related characteristics. Administration times were recorded.

**Results:**

A sample of 153 patients with mild (MMSE 19–24 points; 63%) and moderate (MMSE: 10–18 points; 37%) cognitive impairment was included (age range: 65–99 years; mean MMSE: 19 ± 4, range: 8–24 points; diagnosis of dementia and delirium: 40% and 18%, respectively). Rasch analysis indicated unidimensionality with an overall fit to the model (*P* = 0.107). Internal consistency reliability was excellent (Cronbach’s alpha = 0.92). Eleven out of 13 (85%) hypotheses on construct validity were confirmed. The DEMMI showed good feasibility, and no adverse events occurred. The mean administration time of 5 min (range: 1–10) was not influenced by the level of cognitive impairment. In contrast to some other comparator instruments, no floor or ceiling effects were evident for the DEMMI.

**Conclusions:**

Results indicate sufficient psychometric properties of the DEMMI in older patients with cognitive impairment.

**Trial registration:**

German Clinical Trials Register (DRKS00005591). Registered February 2, 2015.

**Electronic supplementary material:**

The online version of this article (10.1186/s12877-018-0780-9) contains supplementary material, which is available to authorized users.

## Background

Cognitive impairment is common in older people admitted to the acute hospital, with prevalence for dementia estimated to be between 13 and 63% [1, 2], and for delirium to be between 20 and 27% [3, 4].

The International Classification of Functioning, Disability and Health (ICF) defines ‘mobility’ as “moving by changing body position or location or by transferring from one place to another, by carrying, moving or manipulating objects, by walking, running or climbing, and by using various forms of transportation” [5]. Many older acute medical patients with cognitive impairment show mobility limitations, including problems with moving by transferring or by walking. These mobility limitations affect the functional independence and recovery from illness [6]. Therefore, mobility is a key outcome in older people with cognitive impairment that should be assessed frequently [6].

Valid monitoring of mobility alterations in older acute medical patients is challenging [7]. Approximately 30 to 60% of this population cannot stand and walk on hospital admission, leading to significant floor effects of single component measures, such as timed walk tests [6, 8]. Cognitive impairment can further complicate the assessment of mobility, e.g. due to complex test instructions [9, 10].

To overcome such limitations of existing instruments [7], the de Morton Mobility Index (DEMMI) was developed [11]. This performance-based bedside-test has a broad scale width and is quick and simple to administer [11, 12]. There is strong evidence for the DEMMI to be an unidimensional as well as sufficiently construct valid, reliable and responsive measure of older people’s mobility producing interval level scores [11, 13–15]. The aim of this study was to examine the DEMMI’s psychometric properties in older acute medical patients with cognitive impairment.

## Methods

### Design and setting

This cross-sectional study examined the measurement properties of the DEMMI in a consecutive sample of older acute medical patients with dementia, delirium or other cognitive impairment in a geriatric hospital in Cologne, Germany. The study was approved by the Ethical Review board of the University of Cologne and registered a priori (DRKS00005591). Ongoing, written informed consent was provided by all participants. Additional guardian informed consent was approved from all participants with a legal representative and from all participants considered to have limited capability to understand the study procedures. The latter was determined by a consortium composed of the ward physician, the primary nurse and the relatives, if appropriate.

The study used follow-up measures not reported in this paper. Reporting of this study followed the recommendations of the STrengthening the Reporting of Observational studies in Epidemiology (STROBE) statement for cross-sectional studies [16].

### Participants

Participant enrolment was from 4 February to 11 December 2015. We defined 91 screening days which were spread across the study period unsystematically. All acute geriatric inpatients consecutively admitted to the clinic on one of the screening days were assessed for eligibility.

**Fig. 1:**
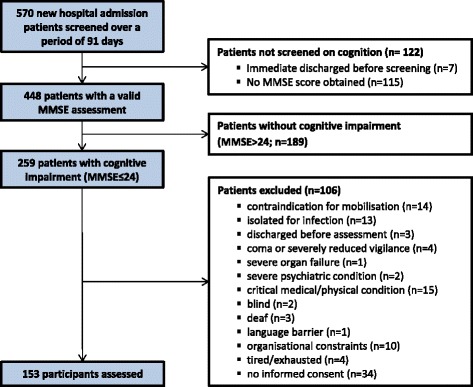
Flow chart of study participants (MMSE: Mini Mental State Examination)

Patients were eligible if they had an unplanned admission to one of the three acute geriatric wards of the hospital, were aged ≥60 years and presented with a cognitive impairment indicated by a Mini Mental State Examination (MMSE) score ≤ 24 points. Exclusion criteria are listed in Fig. 1.

### Procedures

Participants were examined within 7 days after hospital admission by the primary investigator (TB), a physical therapist with 7 years of clinical and academic working experience and well trained in the administration of the outcome measures. In a single session, the DEMMI and a comprehensive set of functional outcomes of mobility were performed in a standardized order, starting with the least physically challenging tests. Socio-demographic data were taken from the medical records and from hospital administrative data. The MMSE [17], the Clock Drawing Test [18] and the 15-item short form of the Geriatric Depression Scale (GDS-15) [19] were assessed by the occupational therapy staff of the hospital as part of routine care (A detailed description of these instruments is given in Additional file 1). Diagnoses and medical symptoms that could be causal for the participant’s cognitive impairment were extracted from the final hospital discharge reports.

### Measurements

The DEMMI is a bedside assessment, consisting of 15 hierarchical mobility items dealing with bed and chair mobility, ambulation, static and dynamic balance [11]. The items are rated with 2-or 3-point response options. The ordinal raw score (0 to 19 points) can be transformed into the interval-level DEMMI score (0 to 100 points). Higher scores indicate better mobility. A German language DEMMI version was used in this study (download free of charge from: www.hs-gesundheit.de) [12, 13].

Table 1 provides an overview of the comparator instruments, including their constructs and scale width. A detailed description of the assessment procedures and a description of the comparator instruments are given in the Additional file 1.

**Table 1:** Construct validity of the de Morton Mobility Index (*n* = 153) including the hypotheses on construct validity and the constructs of the comparison measurement instruments

No.	Hypotheses	Comparison measurement instrument			Observed correlation with DEMMI (Spearman’s correlation)		Hypothesis confirmed
		Measurement instrument	Construct	Mean (SD) or median (IQR)	rho	95% CI	
1	Concerning 1-7, a correlation of > 0.7 was expected between the DEMMI and other broad measures of mobility and walking endurance	Hierarchical Assessment of Balance and Mobility, 0–26 points	Mobility	14 ± 7 (0–26)	0.95	0.93 to 0.96	Yes
2		Performance Oriented Mobility Assessment, 0–28 points	Mobility	7 (1–17)	0.96	0.95 to 0.97	Yes
3		Functional Ambulation Categories, 0–5 points	Ambulation	3 (0–4)	0.92	0.89 to 0.94	Yes
4		Short Physical Performance Battery, 0–12 points	Physical functioning/mobility	1 (0–5)	0.93	0.91 to 0.95	Yes
5		Timed Up and Go test (*n* = 72), sec	Mobility	23 ± 11 (10–63)	0.70	0.56 to 0.80	No
6		Barthel Index mobility subscale, 0–40 points	Mobility	15 (8–30)	0.95	0.93 to 0.96	Yes
7		2-min walk test (*n* = 88), meter	Walking endurance	63 ± 29 (12–126)	0.70	0.58 to 0.79	No
8	Concerning 7-8, a moderate correlation (0.5 < rho ≤ 0.7) was expected between the DEMMI and other single-component mobility scales	4-m walk test (*n* = 85), m/s	Gait speed	0.59 ± 0.23 (0.15–1.15)	0.68	0.55 to 0.78	Yes
9		5× chair rise test (*n* = 28), sec	Lower limb strength	18 ± 6 (9–34)	0.63	0.35 to 0.80	Yes
	Hypotheses	Observed mean DEMMI scores (points) according to clinical groups			Statistical significance (Mann-Whitney U test, 1-fold)		Hypothesis confirmed
		Clinical groups	DEMMI mean score				
10	A statistically significant mean difference between ambulatory (FAC ≥ 3; *n* = 108) participants walking without versus participants walking with a walking aid.	No walking aid (*n* = 21)	67 ± 7 (53–85)		U = 137; *P* < 0.01		Yes
		Walking aid (*n* = 64)	49 ± 11 (27–67)				
11	A statistically significant mean difference between independently ambulatory (FAC ≥ 4) versus dependently ambulatory/non-ambulatory (FAC < 4) participants.	Independent walkers (*n* = 53)	61 ± 9 (39–85)		U = 109; *P* < 0.01		Yes
		Dependent/non-ambulatory (*n* = 100)	27 ± 15 (0–57)				
12	A statistically significant mean difference between participants who can perform the TUG and those who are not able to perform the TUG.	TUG possible (*n* = 72)	56 ± 11 (36–85)		U = 115; P < 0.01		Yes
		TUG not possible (*n* = 81)	22 ± 13 (0–57)				
13	A statistically significant mean difference between participants who can climb stairs and those who cannot.	Able to climb stairs (*n* = 51)	61 ± 9 (39–85)		U = 96; P < 0.01		Yes
		Not able to climb stairs (*n* = 102)	27 ± 15 (0–57)				

*DEMMI* de Morton Mobility Index, *TUG* Timed Up and Go test, *SD* standard deviation, *FAC* Functional Ambulation Categories, *IQR* interquartile-range, *CI* confidence interval

### Statistical analysis

Data were analysed using SPSS 21.0 (IBM Corp.; Armonk, New York), except for the Rasch analysis (RUMM2030 software, version 5.1; www.rummlab.com). Interval-based data were examined for normal distribution with the Shapiro-Wilk test of normality and by visual inspection of the related histograms and p-p-plots. As DEMMI scores were not normally distributed (W = 0.96; *P* < 0.001), non-parametric statistics were applied. *P* < 0.05 indicated statistical significance.

### Measurement properties

#### Rasch analysis/Unidimensionality

The Rasch model is a probabilistic model that asserts that item response is a logistic function of item difficulty and person ability [20]. The DEMMI was developed based on the Rasch model [20] and data fitted the model in various other medical conditions [11, 13–15].

A Rasch analysis was performed to assess the DEMMI’s hierarchical order, internal validity, internal consistency reliability, Differential Item Functioning (or item bias) and logistic item structure. Overall fit of data to the model was deemed acceptable if a set of criteria was fulfilled (Additional file 2). Full details of the process of Rasch analysis are given elsewhere [21, 22]. Reporting followed established recommendations [22]. A target sample size of at least 150 was set to provide 99% confidence within ±0.5 logits [23].

#### Construct validity

In absence of a gold standard for mobility, construct validity was assessed by following the methodological approach of hypotheses testing [24, 25]. A recommended target sample size of at least 100 was set [24].

Aspects of convergent and known-groups validity (functional outcomes and participant’s clinical data) were used to formulate 13 hypotheses a priori (H1 to H13) [24]. All hypotheses were based on existing literature and expertise of clinicians and the research team. Formulated and shortened versions of all hypotheses are given in Additional file 3 and Table 1, respectively.

We applied one-tailed Spearman’s rho analysis because directions of the correlations were hypothesized a priori. For tests in which lower scores represent better functioning (eg. timed up and go test), a negative correlation was hypothesized. All correlations are reported unidirectionally to improve readability. A one-sided Mann Whitney U test was used to compare known groups as directional hypotheses were formulated a priori.

We decided against defining an a priori threshold of a percentage of hypotheses (e.g. 75%) which would need to be confirmed in order for a measurement instrument to be considered valid [25]. Along with others [26], we do not think that the broad concept of construct validity can be judged as “good” or “bad” according to an arbitrary threshold of confirmed hypotheses of varying importance. Instead, we leave it to the reader to decide which percentage of confirmed hypotheses is deemed acceptable.

### Feasibility

Administration times for the DEMMI were recorded for the whole sample and for sub-groups of cognitive impairment severity (MMSE quantiles; two-sided Kruskal Wallis test). Adverse events, such as falls, reports of pain, untypical and severe changes of muscle tone, or significant fatigue were documented. We counted missing values and how often participants had significant difficulty following one or more instructions of the DEMMI due to cognitive impairment.

### Interpretability: Floor and ceiling effects

A floor or ceiling effect was considered if ≥15% of the participants scored the highest or lowest possible DEMMI score or within the minimal detectable change (MDC) of the extremes, respectively [25]. The MDC with 95% confidence (MDC_95_) for the DEMMI in older acute medical patients with cognitive impairment is seven points (based on the present sample; unpublished data to be submitted). Thus, the MDC_95_-adjusted floor and ceiling ranges were 0–7 and 93–100 DEMMI points, respectively.

## Results

A total sample of 153 older acute medical patients with moderate (37%) and mild (63%) cognitive impairment was included in this study (participant flow: Fig. 1; admission characteristics: Table 2). Sixty-one participants (40%) were diagnosed with dementia, 28 (18%) with delirium. Seventy participants (46%) reported a fall and its consequences to be the main reason for hospital admission.

**Table 2:** Characteristics of participants (n = 153)

Characteristic	Value
Age, years	82 ± 7 (65–99)
Gender: male/female, n (%)	54/99 (35/65)
Pre-clinical living situation: home alone/home with family or relatives/ institutionalized, n (%)	89/58/5 (58/39/3)
Total length of stay on the acute ward, days	18 ± 8 (3–64)
Time between admission and assessment, days	3.1 ± 1.6 (0–7)
Primary diagnosis according to ICD-10 categories	
IX Circulatory, n (%)	24 (16)
X Respiratory, n (%)	14 (9)
XI Digestive system, n (%)	7 (5)
XIII Musculoskeletal, n (%)	12 (8)
XVIII Symptoms, signs and abnormal clinical and laboratory findings, not elsewhere classified, n (%)	18 (12)
XIX Injury, poisoning and certain other consequences of external causes, n (%)	52 (34)
Other, n (%)	26 (17)
Potential reasons for cognitive impairment reported in the medical chart (diagnosis, symptom, medical sign; double-counts possible due to multi-morbidity)	
None reported, n	49 (32)
Alzheimer’s dementia, n	7 (5)
Vascular dementia, n	24 (16)
Frontotemporal dementia, n	1 (1)
Dementia, not specified, n	29 (19)
Parkinson’s disease, n	12 (8)
Stroke, n	18 (12)
Depression, n	32 (21)
Delirium, n	28 (18)
Other (psychosis, alcohol abuse, Vitamin B6 deficiency), n	8 (5)
In-hospital walking aid	
Wheeled-walker/rollator, n (%)	60 (39)
None, n (%)	25 (16)
Cane/single crutch, n (%)	12 (8)
Other, n (%)	4 (3)
Non-ambulatory (wheelchair), n (%)	52 (34)
Ambulation	
Independent walkers (FAC ≥4), n (%)	53 (35)
Not ambulatory or dependent walkers (FAC ≤3), n (%)	100 (65)
Barthel Index, 0–100 points	
Valid/missing, n (%)	148/5 (97/3)
Mean score, points (*n* = 148)	46 ± 20 (0–90)
Mini Mental State Examination, 0–30 points, n = 153	
Severe cognitive impairment, 0–9 points, n (%)	1 (1)
Moderate cognitive impairment, 10–18 points, n (%)	56 (37)
Mild cognitive impairment, 19–24 points, n (%)	96 (63)
Mean score, points	19 ± 4 (8–24)
Median score, points	20 (16–22)
Mean time between MMSE and DEMMI assessment, days	2.6 ± 1.6
Median time between MMSE and DEMMI assessment, days	2 (1–4)
Clock Drawing Test, 1–6 points	
Unsuspicious: 1–2 points, n (%)	9 (6)
Suspicious: 3–6 points, n (%)	113 (74)
Missing/not possible, n (%)	31 (20)
Mean score, points (*n* = 122)	4.2 ± 1.2
Geriatric Depression Scale short form, 0–15 points	
Normal: 0–4 points, n (%)	70 (46)
Mild depressive: 5–8 points, n (%)	43 (28)
Moderate depressive: 9–11 points, n (%)	15 (10)
Severe depressive: 12–15 points, n (%)	7 (5)
Missing/not possible, n (%)	3/15 (2/10)
Mean score, points (*n* = 135)	5 ± 3 (0–13)
DEMMI, 0–100 points, points	38 ± 21 (0–85)

*Abbreviations*: *ICD-10* International Classification of Diseases 10th version, *FAC* functional ambulation categories, *DEMMI* de Morton Mobility Index

Values are presented as mean ± standard deviation (range) or median (interquartile range)

There were no missing DEMMI items. The distribution of DEMMI scores is illustrated in the figure in Additional file 4. There was no statistically significant difference in DEMMI scores between subgroups of cognitive impairment (MMSE quantiles; table in Additional file 5), but participants with more severe cognitive impairment tended to have lower DEMMI mean scores than participants in the upper MMSE quartile (35 versus 44 points).

Table 1 includes the mobility related outcomes for all comparator instruments. A substantial proportion of participants could not perform the 2-min walk test (*n* = 65; 42%), the gait speed measure (*n* = 68; 45%) and the five times chair rise test (*n* = 125; 82%) due to physical impairment such as insufficient balance, walking or sit-to-stand transfer abilities. The timed up and go test (TUG) could not be assessed in 81 (53%) participants due to physical impairment (*n* = 78), limited understanding of the test instructions (*n* = 1) and fatigue/refusal after the familiarization trial (*n* = 2).

### Validity

#### Rasch analysis

Rasch analysis was performed on the complete DEMMI item sets of 153 participants. All but ten participants were able to sit unsupported for 10 s (item #4) and no participant was able to perform a tandem stand with eyes closed for 10 s (item #10). Therefore, those two extreme items had to be excluded from the analysis. Further analysis was run on a 13 item DEMMI scale.

Overall fit to the model was achieved with a non-significant (Bonferroni adjusted *P* = 0.05/13 = 0.004) chi-square value (19.54, df = 13, *P* = 0.107). There were no mis-fitting persons and no mis-fitting items as all person and items fit residuals were within ±2.5. There were no disordered thresholds, indicating that the responses to the items are consistent with the metric estimate of the underlying construct of mobility. The Person Separation Index was 0.93 and Cronbach’s alpha was 0.92. Unidimensionality was further confirmed with 6.1% (95% CI: 2.4–9.8) significant independent t-tests at the person level. There was no Differential Item Functioning by sex, age, hospital ward, depression or cognitive impairment, indicating no item bias by any of these factors. Data were confirmed as meeting the assumption of local independence.

**Fig. 2:**
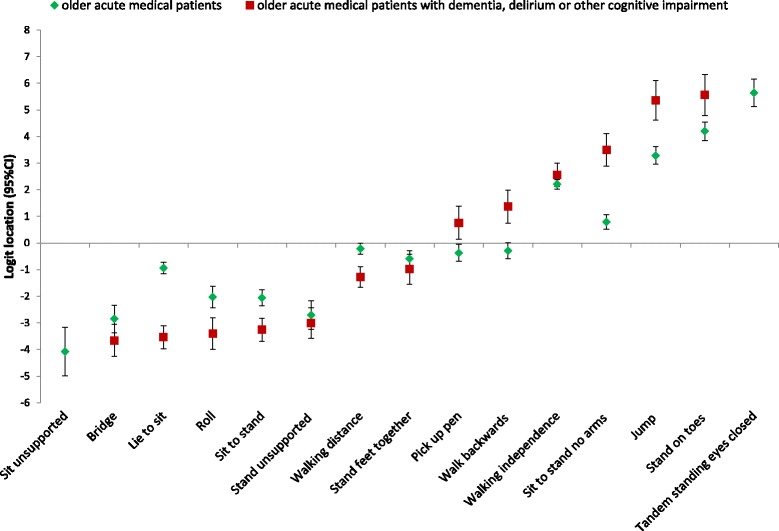
Item logit location: Item logit location (with 95% confidence intervals) and item hierarchy of difficulty of the older acute geriatric sample with dementia or cognitive impairment and the original older acute geriatric sample DEMMI data [11]. Item #4 (sit unsupported for 10 s) and #10 (tandem standing eyes closed) excluded from the dementia analysis. A high positive logit location (e.g. standing on toes) indicates harder item difficulty compared to a negative logit location (e.g. roll). Deviation from the original hierarchy is indicated by non-overlapping 95% confidence bands

The item hierarchy of the DEMMI in the present sample compared to the DEMMI development sample (older acute medical hospitalized patients [11]) is illustrated in Fig. 2. A high positive logit location (e.g. tandem standing eyes closed) indicates harder item difficulty compared to a negative logit location (e.g. bridging). Deviations from the original item hierarchy are indicated by non-overlapping 95% confidence bands in nine items. In the sample of older participants with cognitive impairment, four items were easier (lower logit location: #2 roll, #3 lie to sit, #5 sit to stand, #11 walking distance), and five items were more difficult (higher logit location: #6 sit to stand no arms, #9 stand on toes, #10 jump, #13 pic up pen, #14 walk backwards) than for the aged acute hospitalized sample, with non-overlapping 95% confidence bands.

#### Construct validity

Eleven out of 13 (85%) a priori stated hypotheses about correlations of the DEMMI with other functional measures and known-group differences were confirmed (Table 1).

### Feasibility

The mean administration time of 153 DEMMI assessments was 5.2 ± 2.0 (range: 1–10) minutes. There were no significant differences in administration time between MMSE quantile sub-groups (table in Additional file 5). No adverse events occurred in any DEMMI assessment. We encountered no significant feasibility problems since no participant had difficulty following one or more DEMMI instructions due to cognitive impairment.

### Interpretability - floor and ceiling effects

The figure in Additional file 4 illustrates that neither absolute floor nor ceiling effects occurred, since 10 (7%) participants scored 0 and no participant scored 100 DEMMI points, respectively. Subjected to the MDC_95_-ranges, no floor (7%) or ceiling (0%) effects occurred either.

## Discussion

This study provides first evidence for the DEMMI to be a feasible, unidimensional and construct valid measurement instrument of mobility in older individuals with cognitive impairment, without floor and ceiling effects.

A Rasch analysis confirmed the hierarchical order, internal validity and logistic item structure of the DEMMI already reported for various other geriatric populations [11, 13–15]. Especially evidence for unidimensionality seems clinically important, as clinicians and researchers who use the DEMMI can have confidence to measure the latent trait of mobility. DEMMI items were not biased by the level of cognitive impairment or depression.

The item hierarchy (Fig. 2) indicates that especially transfer items (roll, lie to sit, sit to stand) were easier to achieve for older people with cognitive impairment than for older acute hospitalized participants in the development sample. Most items that were harder to achieve deal with balance abilities (stand on toes, jump, pic up pen, walk backwards). A former comparison [13] between the German DEMMI translation in older sub-acute hospital patients with the Australian aged acute hospitalized DEMMI development population [11] showed non-overlapping 95% confidence bands in five items that followed the same pattern (easier: lie to sit, sit to stand, walking distance; harder: sit to stand no arms, jump). The present deviations from the original item hierarchy might be explained by systematic differences between both language versions of the DEMMI and/or the different sample compositions (46% fall-related acute illnesses in the present sample versus 38% cardio-respiratory principal diagnoses in the development study [11]). A reduced balance is a major risk factor for falls [27]. Because of the high prevalence of participants with fall-related injuries, it is very likely that the present sample included many older people with reduced balance abilities. The deviations from the original item hierarchy does not seem to affect the overall validity of the DEMMI scores in this population based on the results of the Rasch and construct validity analyses, but clinicians should be aware of the altered item hierarchy of the DEMMI in older acute medical patients with cognitive impairment.

The DEMMI showed strong correlations with other established measures of mobility. Especially correlations with multi-component scales that assess mobility apart from walking were all > 0.90. These results indicate good construct validity [24].

There were no floor or ceiling effects on hospital admission, indicating that the DEMMI can be used over the whole mobility spectrum of acute medical patients with dementia, delirium or other cognitive impairment. There is evidence that this is not the case for some of the comparator instruments. In the present study, the walking tests could not be assessed with approximately half of the participants. Rockwood et al. [9] reported 36% of community-dwelling older adults with cognitive impairment to be physically unable to perform the TUG. Such floor effects are even more drastic in representative samples assessed in acute hospitals (admission: 62–74%, discharge: 39–42%) [12, 28].

The DEMMI was performed quicker than usually reported (average 5 versus 8 to 10 min) [11, 12]. Possible explanations might be the high level of routine in DEMMI application by the assessor and the high number of non-ambulatory (34%) participants in the present sample. With these patients, the DEMMI can be performed quickly since the balance and walking items can be omitted according to its hierarchical structure. Administration times of ≤10 min seem realistic in older patients with mild to moderate cognitive impairment, provided that the DEMMI is used by experienced health care professions with a substantial routine in test application. Short administration times of outcome measures facilitate clinical application and enlarge therapy time.

### Limitations

It is possible that sampling bias may exist in the data. We might have missed some potentially eligible patients since we initially excluded 122 (21%) patients without MMSE assessment, caused by organisational constraints, refusal and vigilance issues, among others. We assume a significant number of people with (severe) dementia within this group, especially since it is not unusual that these individuals refuse cognitive assessment [2, 29].

Cognitive impairment of the study sample was indicated by MMSE and Clock Drawing Test results. However, there might be misclassifications, and we cannot causally explain cognitive impairment in all participants. For 60% of participants, there was no formal dementia diagnosis. This is not surprising, since cognitive impairment may be caused by other pathologies or fluctuating acute changes in mental status, such as stroke [30] or delirium [31], respectively. In addition, the diagnosis of dementia can be a time-consuming process that needs longitudinal observation of the course and features of cognitive decline. Usually, it needs to be supported by reports of relatives/carers. This may be difficult in busy acute hospitals with most patients staying for a short time only.

Moreover, in 32% of participants, there was no reasonable pathology/medical sign documented that could explain the cognitive impairment. Many clinicians find it hard to distinguish between dementia and delirium, and it is well known that both are frequently unrecognized and unreported even when present [2, 31, 32]. Further misclassification may be based on patients with depression but intact cognition who scored low on the MMSE [33]. A more detailed psychiatric review of study participants would have helped to even better describe the sample.

The generalisability of the results might be limited as data was collected by one single rater in a single hospital only. Further research should assess the DEMMI’s clinical feasibility when applied by multiple raters in patients with more severe dementia.

## Conclusions

In conclusion, the DEMMI seems to be a unidimensional, construct valid, and reliable performance-based bedside test to measure mobility in older patients with dementia, delirium or other cognitive impairment. The absence of any floor or ceiling effects on hospital admission indicates applicability across the whole mobility spectrum. Further advantages include the short administration time, no need for special equipment, no licence charge, interval level measurement, simple and straightforward items, and an easy scoring system.

Provided that the high feasibility and clinical utility found in this study, and the sufficient reliability and responsiveness of the DEMMI reported for geriatric populations [13, 34, 35] are confirmed in future studies, the DEMMI might become the standard test of mobility in older patients with cognitive impairment. Further, its clinical utility for goal setting and guiding rehabilitation strategies should be explored.

## Additional files

Additional file 1: Detailed description of the assessment procedures and a description of the comparator instruments. (PDF 606 kb)

Additional file 2: Criteria for the Rasch analysis. (PDF 448 kb)

Additional file 3: Formulated hypotheses on construct validity. (PDF 465 kb)

Additional file 4: Histogram of the de Morton Mobility Index. (PDF 394 kb)

Additional file 5: DEMMI mean scores and DEMMI mean administration times for sub-groups of cognitive impairment. (PDF 96 kb)

## Abbreviations

CI: Confidence interval
DEMMI: de Morton Mobility Index
GDS-15: 15-item short form of the Geriatric Depression Scale
MDC_95_: Minimal detectable change with 95% confidence
MMSE: Mini Mental State Examination
TUG: Timed up and go test

## References

[1] Mukadam N, Sampson EL (2011). A systematic review of the prevalence, associations and outcomes of dementia in older general hospital inpatients. Int Psychogeriatr.

[2] Timmons S, Manning E, Barrett A, Brady NM, Browne V, O'Shea E (2015). Dementia in older people admitted to hospital: a regional multi-hospital observational study of prevalence, associations and case recognition. Age Ageing.

[3] Ryan DJ, O'Regan NA, Caoimh RO, Clare J, O'Connor M, Leonard M (2013). Delirium in an adult acute hospital population: predictors, prevalence and detection. BMJ Open.

[4] Whittamore KH, Goldberg SE, Gladman JR, Bradshaw LE, Jones RG, Harwood RH (2014). The diagnosis, prevalence and outcome of delirium in a cohort of older people with mental health problems on general hospital wards. Int J Geriatr Psychiatry..

[5] World Health Organization (2001). International classification of functioning, disability and health: ICF.

[6] Hubbard RE, Eeles EMP, Rockwood MRH, Fallah N, Ross E, Mitnitski A, Rockwood K (2011). Assessing balance and mobility to track illness and recovery in older inpatients. J Gen Intern Med.

[7] de Morton NA, Berlowitz DJ, Keating JL (2008). A systematic review of mobility instruments and their measurement properties for older acute medical patients. Health Qual Life Outcomes.

[8] Fisher S, Ottenbacher KJ, Goodwin JS, Graham JE, Ostir GV (2009). Short physical performance battery in hospitalized older adults. Aging Clin Exp Res.

[9] Rockwood K, Awalt E, Carver D, MacKnight C (2000). Feasibility and measurement properties of the functional reach and the timed up and go tests in the Canadian study of health and aging. J Gerontol A Biol Sci Med Sci.

[10] Sterke CS, Huisman SL, van Beeck EF, Looman CWN, van der Cammen TJM (2010). Is the Tinetti performance oriented mobility assessment (POMA) a feasible and valid predictor of short-term fall risk in nursing home residents with dementia?. Int Psychogeriatr.

[11] de Morton NA, Davidson M, Keating JL (2008). The de Morton mobility index (DEMMI): an essential health index for an ageing world. Health Qual Life Outcomes.

[12] Braun T, Schulz R-J, Hoffmann M, Reinke J, Tofaute L, Urner C (2015). German version of the de Morton mobility index. First clinical results from the process of the cross-cultural adaptation. Z Gerontol Geriatr.

[13] Braun T, Schulz R-J, Reinke J, van Meeteren NL, de Morton NA, Davidson M (2015). Reliability and validity of the German translation of the de Morton mobility index (DEMMI) performed by physiotherapists in patients admitted to a sub-acute inpatient geriatric rehabilitation hospital. BMC Geriatr.

[14] Jans M, Slootweg V, Boot C, de Morton NA, van der Sluis G, van Meeteren NL (2011). Reproducibility and validity of the Dutch translation of the de Morton mobility index (DEMMI) used by physiotherapists in older patients with knee or hip osteoarthritis. Arch Phys Med Rehabil.

[15] de Morton NA, Harding KE, Taylor NF, Harrison G (2013). Validity of the de Morton mobility index (DEMMI) for measuring the mobility of patients with hip fracture during rehabilitation. Disabil Rehabil.

[16] von Elm E, Altman DG, Egger M, Pocock SJ, Gotzsche PC, Vandenbroucke JP (2008). The Strengthening the reporting of observational studies in epidemiology (STROBE) statement: guidelines for reporting observational studies. J Clin Epidemiol.

[17] Folstein MF, Folstein SE, McHugh PR (1975). “Mini-mental state”. A practical method for grading the cognitive state of patients for the clinician. J Psychiatr Res.

[18] Shulman KI (2000). Clock-drawing: is it the ideal cognitive screening test?. Int J Geriatr Psychiatry.

[19] Yesavage JA, Sheikh JI (2008). Geriatric depression scale (GDS) - recent evidence and development of a shorter version. Clin Gerontol.

[20] Rasch G (1980). Probabilistic models for some intelligence and attainment tests.

[21] Pallant JF, Tennant A (2007). An introduction to the Rasch measurement model: an example using the hospital anxiety and depression scale (HADS). Br J Clin Psychol.

[22] Tennant A, Conaghan PG (2007). The Rasch measurement model in rheumatology: what is it and why use it? When should it be applied, and what should one look for in a Rasch paper?. Arthritis Rheum.

[23] Linacre JM (1994). Sample size and item calibration stability. Rasch Measurement Transactions.

[24] de Vet HCW, Terwee CB, Mokkink LB, Knol DL (2011). Measurement in medicine: a practical guide.

[25] Terwee CB, Bot SDM, de Boer MR, van der Windt DAWM, Knol DL, Dekker J (2007). Quality criteria were proposed for measurement properties of health status questionnaires. J Clin Epidemiol.

[26] Reeve BB, Wyrwich KW, Wu AW, Velikova G, Terwee CB, Snyder CF (2013). ISOQOL recommends minimum standards for patient-reported outcome measures used in patient-centered outcomes and comparative effectiveness research. Qual Life Res.

[27] Rubenstein LZ (2006). Falls in older people: epidemiology, risk factors and strategies for prevention. Age Ageing.

[28] Dasenbrock L, Berg T, Lurz S, Beimforde E, Diekmann R, Sobotka F, Bauer JM (2016). The De Morton mobility index for evaluation of early geriatric rehabilitation. Z Gerontol Geriatr.

[29] Boustani M, Perkins AJ, Fox C, Unverzagt F, Austrom MG, Fultz B (2006). Who refuses the diagnostic assessment for dementia in primary care?. Int J Geriatr Psychiatry..

[30] Mijajlovic MD, Pavlovic A, Brainin M, Heiss W-D, Quinn TJ, Ihle-Hansen HB (2017). Post-stroke dementia - a comprehensive review. BMC Med.

[31] Fong TG, Davis D, Growdon ME, Albuquerque A, Inouye SK (2015). The interface between delirium and dementia in elderly adults. Lancet Neurol.

[32] Inouye SK, Foreman MD, Mion LC, Katz KH, Cooney LM, JR. (2001). Nurses’ recognition of delirium and its symptoms: comparison of nurse and researcher ratings. Arch Intern Med.

[33] Downing LJ, Caprio TV, Lyness JM (2013). Geriatric psychiatry review: differential diagnosis and treatment of the 3 D's - delirium, dementia, and depression. Curr Psychiatry Rep.

[34] de Morton NA, Davidson M, Keating J (2010). Validity, responsiveness and the minimal clinically important difference for the de Morton mobility index (DEMMI) in an older acute medical population. BMC Geriatr.

[35] de Morton N, Davidson M, Keating JL (2010). Reliability of the de Morton mobility index (DEMMI) in an older acute medical population. Physiother Res Int.

