# Supplementary material for: Measuring mobility in older hospital patients with cognitive impairment using the de Morton Mobility Index
Source: BMC Geriatr. 2018 Apr 23;18:100. doi: 10.1186/s12877-018-0780-9 (PMC5913915; doi:10.1186/s12877-018-0780-9)
Supplement: Supplementary file 2 — Criteria for the Rasch analysis. (PDF 448 kb) [file 12877_2018_780_MOESM2_ESM.pdf]

Additional file 2: Criteria for the Rasch analysis

The unrestricted (partial credit) Rasch polytomous model was used with a conditional pair-wise parameter estimation and two class intervals. Overall fit of data to the model is deemed acceptable if the following criteria are fulfilled (adopted from Mills et al. [1]):

(1) ordered item category thresholds;
(2) both total chi-square probability and individual item chi-square probability values non-significant (5% alpha with Bonferroni correction for the number of items);
(3) individual item fit residual, by convention, within ±2.5;
(4) mean and SD of both item fit residual and person fit residual approaching 0 and 1 respectively;
(5) person-item separation index (PSI) (reliability) greater than 0.70 for group use and 0.85 for individual use;
(6) Differential Item Functioning (DIF) occurs when different groups within the sample (e.g., women and men) respond in a different manner to an individual item, despite equal levels of the underlying characteristic being measured. ANOVA probability for differential item functioning (DIF) non-significant (5% alpha with Bonferroni correction) for the following factors: sex, age (split at median: 65 to 82 years and 83+ years), ward (2 normal wards and 1 ward for patients with a private health insurance), cognitive impairment by MMSE (split at median: 0-20 and 21-24 points), cognitive impairment by Clock Drawing Test (split at median: 1-2 and 3-6 points), and by depression (split at GSD-15 median: 0-4 and 5-15 points). This is undertaken with a two way ANOVA with class interval (grouped level of mobility) and the external factor (e.g. sex) as main effects. Uniform DIF is then for the main effect of the factor (e.g. gender; and there is another for class interval) and non-uniform DIF is the interaction between class interval and the factor. For DIF analysis, clinical meaningful groups of approximately equal sizes are needed.
(7) Unidimensionality (all items reflecting a single underlying latent trait) by independent t-test at the person abilities showing less than 5% of tests to be significant or the lower bound of a binomial 95% confidence interval of the observed proportion overlaps 5% [2].
(8) Pearson correlation coefficients between item residuals less than 0.3 (local independence). If the correlation was >0.3, a subtest analysis using the correlated items was undertaken to determine whether the internal consistency (PSI and Cronbach alpha) of the whole item set was higher than for the subtest.

References Additional file 2

1. Mills RJ, Pallant JF, Koufali M, Sharma A, Day S, Tennant A, Young CA. Validation of the Neurological Fatigue Index for stroke (NFI-Stroke). Health Qual Life Outcomes. 2012;10:51.
2. Tennant A, Pallant JF. Unidimensionality Matters! (A Tale of Two Smiths?). Rasch Measurement Transactions. 2006;20:1048–51.
