# Supplementary material for: Measuring mobility in older hospital patients with cognitive impairment using the de Morton Mobility Index
Source: BMC Geriatr. 2018 Apr 23;18:100. doi: 10.1186/s12877-018-0780-9 (PMC5913915; doi:10.1186/s12877-018-0780-9)
Supplement: Supplementary file 3 — Formulated hypotheses on construct validity. (PDF 465 kb) [file 12877_2018_780_MOESM3_ESM.pdf]

Additional file 3: Formulated hypotheses on construct validity

- H1-6: De Morton Mobility Index (DEMMI) scores correlate strongly (>0.7) with other broad measures of mobility [Hierarchical Assessment of Balance and Mobility (HABAM), Performance Oriented Mobility Assessment (POMA), Functional Ambulation Categories (FAC), Short Physical Performance Battery (SPPB), Timed Up and Go test (TUG), mobility subscale of the Barthel Index), as reported by others [1–3].
- H7: DEMMI scores correlate strongly (>0.7) with the 2-minute walk test, a measure of walking capacity. De Morton et al [4] found rho=0.76 between DEMMI and the 6-minute walk test in older patients with hip fracture.
- H8: DEMMI scores correlate moderately (0.5<rho≤0.7) with gait speed, a single component mobility measure. De Morton et al. [4] found rho=0.62 between DEMMI scores and gait speed of older patients with hip fracture.
- H9: DEMMI scores correlate moderately (-0.5<rho≤-0.7) with the 5 times chair rise test, a single component mobility measures. Jans et al. [2] found rho=-0.63 between DEMMI and 5 times chair rise test in in older patients with knee or hip osteoarthritis.
- H10: Ambulatory participants (FAC≥3) ambulating without a walking aid have higher DEMMI scores than participants using a walking aid.
- H11: Independent walkers (FAC≥4) have higher DEMMI scores than non-ambulatory participants or dependent walkers (FAC≤3).
- H12: Participants who are able to perform the TUG (capturing basic mobility aspects) have higher DEMMI scores than participants who cannot perform the TUG.
- H13: The stair item of the Barthel Index assesses the ability to climb 1 flight of stairs, up and down, with the use of a walking aid and the handrail if needed. We expected participants who scored 5 or 10 points (stand-by assistance or independent, respectively) and those who scored 0 points (not able) on this item to show a difference in mean DEMMI scores.

References Additional file 3

1. de Morton NA, Davidson M, Keating JL. The de Morton Mobility Index (DEMMI): an essential health index for an ageing world. Health Qual Life Outcomes. 2008;6:63.
2. Jans M, Slootweg V, Boot C, de M, van d, van M. Reproducibility and validity of the Dutch translation of the de Morton Mobility Index (DEMMI) used by physiotherapists in older patients with knee or hip osteoarthritis. Arch Phys Med Rehabil. 2011;92:1892–9.
3. Braun T, Schulz R-J, Reinke J, van Meeteren NL, Morton NA de, Davidson M, et al. Reliability and validity of the German translation of the de Morton Mobility Index (DEMMI) performed by

physiotherapists in patients admitted to a sub-acute inpatient geriatric rehabilitation hospital. BMC Geriatr. 2015;15:1660.

4. de Morton NA, Harding KE, Taylor NF, Harrison G. Validity of the de Morton Mobility Index (DEMMI) for measuring the mobility of patients with hip fracture during rehabilitation. Disabil Rehabil. 2013;35:105–11.
