# Supplementary material for: Measuring mobility in older hospital patients with cognitive impairment using the de Morton Mobility Index
Source: BMC Geriatr. 2018 Apr 23;18:100. doi: 10.1186/s12877-018-0780-9 (PMC5913915; doi:10.1186/s12877-018-0780-9)
Supplement: Supplementary file 4 — Histogram of the de Morton Mobility Index. (PDF 394 kb) [file 12877_2018_780_MOESM4_ESM.pdf]

## Additional file 4: Histogram of the de Morton Mobility Index

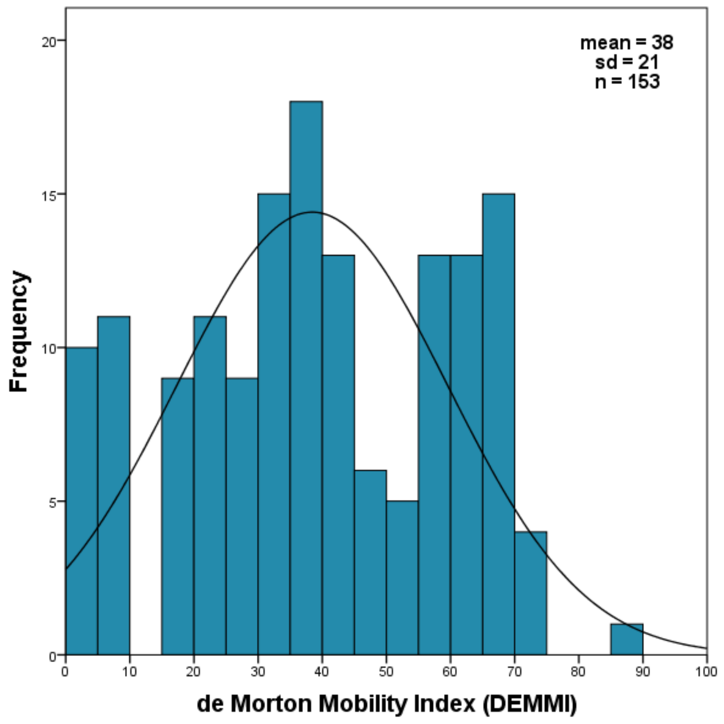


*Histogram of the de Morton Mobility Index scores (range: 0 - 100 points) of all study participants (153 geriatric patients with cognitive impairment)*
