# Supplementary material for: Measuring mobility in older hospital patients with cognitive impairment using the de Morton Mobility Index
Source: BMC Geriatr. 2018 Apr 23;18:100. doi: 10.1186/s12877-018-0780-9 (PMC5913915; doi:10.1186/s12877-018-0780-9)
Supplement: Supplementary file 5 — DEMMI mean scores and DEMMI mean administration times for sub-groups of cognitive impairment. (PDF 96 kb) [file 12877_2018_780_MOESM5_ESM.pdf]

Additional file 5: DEMMI mean scores and DEMMI mean administration times for sub-groups of cognitive impairment

DEMMI mean scores and DEMMI mean administration times for sub-groups of cognitive impairment				
			DEMMI points, mean score ± SD (range)	DEMMI administration time, mean score ± SD (range)
Full sample			38 ± 21 (0 – 85)	5.2 ± 2.0 (1 – 10)
Mini Mental State Examination sub-groups				
Quantile	Range	Frequency (%)		
First	0 – 16 points	42 (27)	35 ± 22 (0-67)	5.0 ± 2.3 (1 – 9)
Second	17 – 20 points	39 (25)	36 ±21 (0 – 74)	5.2 ± 2.2 (2 – 10)
Third	21 – 22 points	35 (23)	40 ± 20 (8 – 85)	5.1 ± 1.7 (2 – 9)
Forth	23 – 24 points	37 (24)	44 ± 22 (0 – 74)	5.3 ± 1.9 (2 – 9)
			Chi^2=3.67; p=0.30	Chi^2=0.70; p=0.87
Abbreviations: DEMMI = de Morton Mobility Index; SD = standard deviation				
